# Editorial

**Published:** 1870-05

**Authors:** 


### Editorial.

Illinois State Medical Society.—This Society holds its next regular annual meeting at Dixon, on the *third Tuesday* in May. The local profession are making arrangements to give members of the Society a very hospital reception.

We are informed by the Permanent Secretary, that he has made arrangements with the Illinois Central, the North-Western, Burlington and Quincy, Rock-Island, and Chicago, Alton, and St. Louis railroads, to carry all members of the State Society to *Dixon*, and return, for one full fare and one-fifth. All who wish to avail themselves of this arrangement should apply to the ticket agents, when they start, for excursion tickets to Dixon and return.

Chicago Medical Society.—At the annual meeting of this Society, held during the first week in April, delegates were appointed to attend the American Medical Association, which meets in the City of Washington, on Tuesday, the 3d day of May, and the State Medical Society, that assembles in Dixon, on the third Tuesday in May.

The following officers were chosen for the ensuing year:—

*President*, Dr. T. D. Fitch; *Vice-President*, F. A. Emmons; *Secretary and Treasurer*, Dr. C. C. Dumreicher.

The regular meetings of the Society are held on Monday evening of each week.

The New Mercy Hospital.—The spacious and elegant new hospital building, on the corner of Calumet Avenue and Twenty-sixth Street, is now completed, and some of its rooms already occupied by the sick. The location is one of the most pleasant and healthy in the City of Chicago, and easy of access. It is one of the best finished and best apportioned buildings for a hospital that we have ever seen. The amphitheatre is elegantly lighted, and will seat comfortably a class of 300 students. The public wards are light, cheerful, and susceptible of perfect ventilation. The small or private wards, designed to accommodate but one or two patients, are numerous, and, in all respects, as pleasant as the rooms of a first-class hotel. It is, in the most proper sense, a general hospital, arranged into three departments, medical, surgical, and obstetrical, including diseases of women. The first is under the charge of Professors N. S. Davis and H. A. Johnson; the second under Professor E. Andrews; and the third under Professor W. H. Byford. The entire domestic or internal management is in the hands of the Sisters of Mercy. The cost of the new edifice is not far from $100,000; and we know of no more comfortable place for the sick, outside of their own families, in this country.

---

CHICAGO MEDICAL COLLEGE.—The work on the new edifice, for the accommodation of this institution, is progressing rapidly, and will be complete in every part before the opening of the next annual lecture term, on the first Monday in October.

It occupies a part of the same lot on which the new Mercy Hospital stands; and, when completed, the hospital and college together, all *professionally* under the control of the college Faculty, and subject to no interference by municipal or political influences, will constitute the most complete and comprehensive arrangement for medical instruction, in all departments, that exists in this country. It being the only college in the whole country which, up to this time, has had the boldness to adopt and put in practice, the system of progressive medical education, by requiring some preliminary education, three consecutive courses of lectures, of five or six months each, with close examinations at the close of each, and full hospital clinical instruction, as an essential part of the middle and senior courses, we deem its prosperity a matter of gratification to the whole profession. The class at present in attendance on the summer reading and clinical course is an excellent one, and everything looks fair in the future.

---

NEW MEDICAL JOURNAL.—We cheerfully call the attention of our readers to the following prospectus of the *National Medical Journal:*—

In presenting another candidate for public favor, in the form of a medical journal, the publishers are not insensible to the large number of valuable periodicals already claiming a share of professional patronage. The field of labor, however, is broad, and much of practical utility may be gleaned in the path of more experienced reapers. Medicine, in all its departments, was never more prolific of great practical results than at present; the medical mind never more vigorous and prehensile in the pursuit of truth.

There appears to be a peculiar appropriateness in locating a first-class medical journal in Washington City, not only on account of its central position, and the various important interests clustering in and around it, but because it embraces a good share of professional ability, needing only the stimulus to medico-literary effort, which a judicious, liberal, and well-conducted periodical affords.

While the principal object of the journal will be to originate and collate useful information in the various branches of medical and surgical science, it is not intended to ignore those prominent *specialties* which have enlisted the zeal and skill of not a few able and enlightened American practitioners. These cannot much longer occupy a subordinate place in the academies and journals of the country; and it is proposed, therefore, to encourage their cultivation and practice within prescribed professional limits.

The journal will be under the editorial management of C. C. Cox, M.D., LL.D., Professor of Anatomy in Georgetown Medical College, and no pains will be spared to render it useful to the medical public, whose patronage and support it seeks.

Each number will consist of not less than 128 pages, octavo, printed in distinct type, on fine paper, with good, substantial covers, and (for the present) will be published quarterly.

Terms: $3 a year, invariably in advance; single copies mailed to any address for $1.

All contributions intended for the journal, advertisements, or subscriptions should be addressed to Judd & Detweiler, Publishers, Corner of Pennsylvania Avenue and 11th St., Washington, D.C.

### Mortality for the Month of March, 1870.

CAUSES OF DEATH.

| | | | | | |
|---|---|---|---|---|---|
| Accident, machinery | 2 | Dropsy | 3 | Lungs, hemorrhage | 2 |
| " by fall | 4 | " of abdomen | 1 | Mouth canker-sore | 1 |
| " fall of coal | 1 | Dysentery | 2 | Measles | 17 |
| " fracture of skull | 1 | " chronic | 1 | " & complications | 10 |
| " thro'n fr'm bug'y | 1 | Ecthyma | 1 | Metro-peritonitis | 2 |
| " railroad | 1 | Encephalitis | 1 | Meningitis | 8 |
| Abscess, renal | 1 | Endo-pericarditis, | | " and dysentery | 2 |
| Albuminuria | 1 | rheumatic, | 1 | " cerebro-spinal | 1 |
| Anæmia and dysentery | 1 | Enteritis | 5 | " tubercular | 8 |
| Anasarca | 1 | Epilepsy | 3 | Nephritis | 1 |
| Angina pectoris, | 1 | Erysipelas | 5 | Old age | 17 |
| Apoplexy | 2 | " of face | 1 | Ophthalmia | 1 |
| Arachnitis | 1 | Fever, puerperal | 8 | Paralysis | 1 |
| Ascitis | 1 | " remittent | 2 | Pericarditis | 1 |
| Asphyxia | 2 | " scarlet | 32 | Peritonitis | 2 |
| Asthma | 3 | " " complications | 6 | Pleurisy, chronic | 1 |
| Births, still | 39 | " " malignant | 14 | Pneumonia | 39 |
| " premature | 26 | " typhoid | 16 | " & complications | 2 |
| Bowels, hemorrhage | 1 | Gastritis | 2 | " broncho | 4 |
| " ulceration | 1 | Gastro-enteritis | 1 | " typhoid | 3 |
| Brain, abscess | 1 | Heart disease | 3 | Pyæmia | 3 |
| " congestion of | 2 | " and liver, fatty | | Rheumatism, chronic | 1 |
| " compression | 1 | degeneration | 1 | " inflammatory | 1 |
| " inflammation | 2 | " hypertrophy | 1 | Scrofula | 1 |
| Bronchitis | 12 | " organic disease, | 2 | Stomach, cancer | 3 |
| " capillary | 8 | " valvular disease | 5 | Suicide, hanging | 1 |
| Biliary calculi | 1 | Hernia, incarcerated | 1 | " poison | 1 |
| Breast, cancer | 2 | " strangulated | 1 | " shooting | 1 |
| Catarrh | 1 | Hydrocephalus | 3 | Tabes mesenterica, | 18 |
| Consumption | 46 | " acute | 6 | Teething | 4 |
| Convulsions | 53 | " chronic | 1 | " & complications | 3 |
| " puerperal | 2 | Inanition | 10 | Tetanus following com- | |
| Croup | 16 | Ichorrhæmia | 1 | poud fracture of leg | 1 |
| " diphtheretic | 1 | Jaundice | 1 | Throat, canker-sore | 1 |
| " membranous | 3 | Kidneys, Bright's dis- | | Toxæmia | 1 |
| Cystitis, | 1 | ease of | 4 | Tumor of abdomen | 1 |
| Cyanosis | 2 | " disease | 1 | Uræmia | 1 |
| Cynanche Maligna | 1 | Laryngitis | 3 | Uterus, hemorrhage | 1 |
| Debility | 4 | Liver, abscess | 2 | Whooping-cough | 11 |
| Diabitis | 1 | " hypertrophy | 1 | " & complications | 1 |
| Diarrhœa | 1 | " inflammation and | | Unknown | 1 |
| " chronic | 1 | peritonitis | 1 | | |
| Diphtheria | 11 | Lungs, congestion | 4 | Total | 535 |

AGES.

| | | | | | |
|---|---|---|---|---|---|
| Under 1 | 173 | 10 to 20 | 21 | 70 to 80 | 18 |
| 1 to 2 | 67 | 20 to 30 | 42 | 80 to 90 | 6 |
| 2 to 3 | 42 | 30 to 40 | 36 | Unknown | 1 |
| 3 to 4 | 21 | 40 to 50 | 26 | | |
| 4 to 5 | 16 | 50 to 60 | 17 | Total, | 535 |
| 5 to 10 | 28 | 60 to 70 | 21 | | |

| | | | | | |
|---|---|---|---|---|---|
| Males, | 288 | Females, | 247 | Total, | 535 |
| Single, | 403 | Married | 132 | Total, | 535 |
| White, | 525 | Colored, | 10 | Total, | 535 |

COMPARISON.

| | | |
|---|---|---|
| Deaths in March, 1870, 535 | Deaths in March 1869, 354 | Increase, 181 |
| Deaths in Feb., 1870, | 420 | Increase, 115 |

NATIVITY.

| | | | | | |
|---|---|---|---|---|---|
| Bohemia | 4 | Germany | 54 | Sweden | 4 |
| Canada | 4 | Holland | 1 | Switzerland | 2 |
| Chicago, Native | 92 | Ireland | 46 | Wales | 1 |
| Chicago, Foreign | 221 | Norway | 6 | Unknown | 1 |
| U. S., other parts | 82 | Poland | 1 | | |
| Denmark | 4 | Scotland, | 3 | Total, | 535 |
| England | 9 | | | | |

MORTALITY BY WARDS FOR THE MONTH.

| Wards. | Mortality. | | Mortality. |
|---|---|---|---|
| 1 | 2 | Accidents | 10 |
| 2 | 15 | County Hospital | 12 |
| 3 | 22 | Home for Friendless | 5 |
| 4 | 16 | Hospital Alexian Brothers | 2 |
| 5 | 19 | Half Orphan Asylum | 4 |
| 6 | 23 | Immigrants | 1 |
| 7 | 27 | Marine Hospital | 1 |
| 8 | 42 | Jewish Hospital | 1 |
| 9 | 48 | Mercy Hospital, | 7 |
| 10 | 8 | Protestant Orphan Asylum | 2 |
| 11 | 27 | St. Joseph Orphan Asylum, | 5 |
| 12 | 19 | Soldiers' Home | 2 |
| 13 | 11 | Suicides | 3 |
| 14 | 15 | St. Louis and Chicago R.R. Dépôt | 1 |
| 15 | 32 | | |
| 16 | 25 | Total, | 535 |
| 17 | 36 | | |
| 18 | 43 | | |
| 19 | 17 | | |
| 20 | 22 | | |

THE SALE OF "BITTERS."—The consumption of various stomach bitters is one of the most common, insidious, and perilous forms of tippling. Many a man and many a woman have learned indulgence from this cause. We are glad to see that the Supreme Judicial Court of Massachusetts has recently made an important decision in a prosecution arising from the sale of a bottle of bitters, in which the defendant asserted that he has sold the liquid in good faith as a medicine. The judge trying the case, however, charged the jury that if the "bitters" was an intoxicating liquor, the sale of it in good faith as a medicine was not a valid defence, and this position was maintained by the Supreme Judicial Court. As a consequence of this decision the State Constables are seizing "bitters" under the Massachusetts law against the sale of intoxicating liquors.—*Medical and Surgical Reporter.*

Nephrotomy.—On Thursday, Feb. 3, the operation of cutting into the kidney for removal of renal calculus was performed by Mr. Durham at Guy's Hospital. The theatre was crowded with students and others anxious to see so rare an operation. We believe, indeed, that there is only one instance of its performance recorded, and that was by a surgeon of Venice upon an Englishman, some 150 years since. Two or three stones where then extracted, to the great relief of the patient, a urinary fistula remaining in the loin. Owing, doubtless, as much to the uncertainty of the diagnostic signs of renal calculus as to the supposed risks of the procedure, the operation fell into oblivion, until a paper read before the Medico-Chirurgical Society last year, by Mr. Thomas Smith, of St. Bartholomew's Hospital, again brought the subject before the profession. Although Mr. Durham's bold attempt ended in disappointment, we saw enough to convince us that, as far as cutting down upon the kidney is concerned, there is neither great difficulty nor any apparent grave risk in the proceeding. Mr. Durham made his incision along the edge of the erector spinæ, from the pelvis to the eleventh rib, and quickly reached the hilus of the without difficulty, and with little or no loss of blood, but no stone was found, although the symptoms previously manifested had been such as are considered characteristic of stone in the kidney. The hilus of the kidney and the ureter, for the space of an inch and a-half, were thoroughly examined, but not opened, no stone being felt and their general appearance, as well as that of the kidney, being perfectly healthy. So far from the operation having been injurious, five days later the woman expressed herself as being more free from pain than she had been for a long time! Full details of the case will be published whenever it may be considered to have terminated, whatever the termination may be.—*Medical Times & Gazette.*

---

Prurigo Treated by Ointment of Iodoform.—Prof. Tanturri, of Naples, has used the ointment of iodoform in obstinate prurigo. This compound, first brought prominently into notice by Bouchardat, is now employed extensively not only for glandular enlargements, but also, owing to its anæsthetic properties, in skin diseases accompanied with intense pruritus; its odor is much more agreeable than that of chloroform, resembling that of safron. Moretin and Humbert recommend it for internal use as possessing all the advantages of iodine, of which it contains 90 per cent., without any of its inconveniences. It exercises upon the sphincters a local anæsthetic effect so powerful that defecation is sometimes performed unconsciously after its use; it, therefore, forms an admirable suppository in cases of tenesmus, hæmorrhoids, etc. Moûtre's formula is—iodoform, powdered, gr. xx; cocoa butter, ʒj; melt and mix for six suppositories. For frictions, the ointment is used in the strengh of ʒj to the ounce of simple ointment.—*Med. Times & Gaz.*

